# Zinc Absorption from Micronutrient Powder Is Low but Is not Affected by Iron in Kenyan Infants

**DOI:** 10.3390/nu6125636

**Published:** 2014-12-08

**Authors:** Fabian Esamai, Edward Liechty, Justus Ikemeri, Jamie Westcott, Jennifer Kemp, Diana Culbertson, Leland V. Miller, K. Michael Hambidge, Nancy F. Krebs

**Affiliations:** 1 Moi University School of Medicine, P.O. Box 4606, Eldoret 30100, Kenya; E-Mails: fesamai2007@gmail.com (F.E.); jusmeri06@yahoo.co.uk (J.I.); 2 Indiana University School of Medicine, 705 Riley Hospital Drive, Room 5900, Indianapolis, IN 46202, USA; E-Mail: eliecht@iupui.edu; 3 Section of Nutrition, Department of Pediatrics, School of Medicine, University of Colorado, Denver, Aurora, CO 80045, USA; E-Mails: jamie.westcott@ucdenver.edu (J.W.); jennifer.kemp@ucdenver.edu (J.K.); dianaculbertson@gmail.com (D.C.); leland.miller@ucdenver.edu (L.V.M.); michael.hambidge@ucdenver.edu (K.M.H.)

**Keywords:** zinc absorption, micronutrient powders, iron supplementation, malaria, Kenya

## Abstract

Interference with zinc absorption is a proposed explanation for adverse effects of supplemental iron in iron-replete children in malaria endemic settings. We examined the effects of iron in micronutrient powder (MNP) on zinc absorption after three months of home fortification with MNP in maize-based diets in rural Kenyan infants. In a double blind design, six-month-old, non-anemic infants were randomized to MNP containing 5 mg zinc, with or without 12.5 mg of iron (MNP + Fe and MNP − Fe, respectively); a control (C) group received placebo powder. After three months, duplicate diet collections and zinc stable isotopes were used to measure intake from MNP + non-breast milk foods and fractional absorption of zinc (FAZ) by dual isotope ratio method; total absorbed zinc (TAZ, mg/day) was calculated from intake × FAZ. Mean (SEM) TAZ was not different between MNP + Fe (*n* = 10) and MNP − Fe (*n* = 9) groups: 0.85 (0.22) and 0.72 (0.19), respectively, but both were higher than C (*n* = 9): 0.24 (0.03) (*p* = 0.04). Iron in MNP did not significantly alter zinc absorption, but despite intakes over double estimated dietary requirement, both MNP groups’ mean TAZ barely approximated the physiologic requirement for age. Impaired zinc absorption may dictate need for higher zinc doses in vulnerable populations.

## 1. Introduction

Iron deficiency and iron deficiency anemia are among the most prevalent and intractable public health problems in infants and young children, particularly in malaria-endemic, resource-limited settings. However, oral iron supplementation has been associated with increased mortality and rates of adverse events from infectious diseases in older iron-replete infants and toddlers in a malaria endemic area [1]. These adverse outcomes have prompted critical re-evaluation of approaches to prevent iron deficiency and iron deficiency anemia [2,3]. There is an urgent need to identify the mechanisms of such untoward effects of iron administration, since halting population-based initiatives in the face of widespread deficiency is not an option.

Interference with zinc absorption and/or metabolism is one proposed explanation for the adverse effects of iron supplements in iron replete subjects in malaria endemic areas [2]. Evidence for an interaction between iron and zinc is conflicting [4,5], and actual mechanisms are not clear. Iron supplements have been associated with a reduction in plasma zinc concentrations [6,7,8]. In two studies of lactating women, iron supplementation was associated with lower fractional absorption of zinc [9,10]. Studies in pregnant women have reported conflicting results for an effect of iron supplements on zinc absorption [11,12]. Neither iron added to food through fortification, nor supplemental iron when ingested with meals has been found to have an adverse effect on zinc absorption in healthy subjects [13,14]. A recently published mathematical model of zinc absorption in adults found no effect of iron on zinc absorption when calcium and protein intakes were controlled for [15]. Studies examining this issue in young children in low resource settings, however, are limited.

For this study, we examined in a vulnerable population the effects on zinc absorption of increased iron intake by comparing micronutrient powder (MNP) with and without iron, compared to a study group receiving placebo MNP as a control. The setting for the study was rural Kenya, where iron deficiency is high in older infants and toddlers; local diets for complementary feeding are typically monotonous, plant based foods low in iron and zinc and high in phytate; and malaria is endemic.

The objective was to determine the effects of iron in MNP on zinc absorption and the size of the exchangeable zinc pool (EZP), as an indicator of zinc status, after three months of home fortification with MNP in maize-based diets. We hypothesized that there would be a significantly lower absorption of zinc in the MNP with iron compared to that of the infants receiving MNP without iron.

## 2. Experimental Section

### 2.1. Study Design

The study was a randomized, double blinded comparison of total daily zinc absorption in 15 infants (9 months of age) from each of three MNP groups: one with multiple micronutrients, including 5 mg zinc and 12.5 mg iron (MNP + Fe); another with same composition except no iron (MNP − Fe); and control with placebo sachets including no micronutrients (C). The daily MNP intervention was started at 6 months of age, when all subjects were predominantly breastfed (no formula). All meals for a day were extrinsically labeled with one of two stable isotopes of zinc; ^67^Zn was administered with the meal containing the MNP sachet, and ^70^Zn was used to label other meals of the day. An intravenous dose of a third stable isotope of zinc (^68^Zn) was administered to enable measurement of zinc absorption by dual isotope tracer ratio (DITR) in urine method [16]. In conjunction with the intravenous isotope administration, blood was drawn for biomarkers of iron and zinc status. The size of the EZP was also determined from the enrichment of ^68^Zn in urine [17].

### 2.2. Study Setting

The study was undertaken in rural western Kenya, approximately 150 km from Moi University Medical School in Eldoret. The villages are characterized by extreme poverty, with 40%–80% of the population living below the World Bank defined poverty level. The predominant occupation is subsistence farming. Most families live in traditional homes with mud walls, dirt floors, thatched roofs; virtually none had in-home electricity or public water source. The area is holoendemic for *P. falciparum* malaria. The traditional diets are monotonous, predominantly maize-based. For the infants participating in the study, the diets were primarily maize-based porridge mixed with breast milk; diets also included some ugali (maize), vegetables, and occasional meat soups.

A convenience sample of 45 infants was drawn from participants in the Maternal and Neonatal Registry study of the Global Network for Women’s and Children’s Health [18]. Families who had successfully participated in the registry were approached for participation in the MNP supplementation and zinc absorption study when the infants were approximately 5 months old. The registry administrators of this cluster were requested to provide a list of 45 consecutive infants at this age from their registers for inclusion into the study based on the study inclusion criteria. Infants drawn from only two of the 39 villages in the cluster were selected for ease of follow up. The infants’ homes were visited by the study field staff accompanied by the registry administrator for purposes of identification.

### 2.3. Subjects

Subjects were enrolled just prior to 6 months of age and received the randomly assigned daily MNP until 9 months of age. Inclusion criteria included the infant having been born at term with birth weight > 2500 g; healthy appearance with no apparent congenital anomalies; current immunization status; family social situation such that they were able to comply with study requirements; willingness to provide informed consent and agree to weekly visits for MNP distribution and data collection, as well as stool, urine and blood collections; hemoglobin concentration ≥ 10 g/dL; infant still breastfeeding at enrollment and intended to continue during the three months of the study period; and negative blood slide for malaria parasites. Exclusion criteria included existing acute malnutrition, especially wasting, with weight for length ≤ 2 SD by World Health Organization (WHO) infant growth standards; current or planned use of infant formula or other nutrient fortified products; current or planned use of iron or zinc supplements; anemia (defined as Hb < 10 g/dL); previous hospitalization for malaria within the last four weeks; and persistent diarrhea (per WHO, >3 loose stools/day lasting ≥14 days). Infants’ stools were screened for helminthes; none were positive for infestation. Approval from the institutional and ethics review committees at Moi University School of Medicine and the University of Colorado was obtained prior to study implementation. The study is registered in ClinicalTrials.gov NCT02101723.

Sample size calculations were based on results of infant zinc absorption studies previously conducted in the University of Colorado Denver (UCD) laboratory [19,20]. With the use of independent sample *t*-tests, we used PASS software (NCSS—Number Cruncher Statistical Systems 2007 and PASS 2008; Number Cruncher Statistical Systems, Kaysville, UT, USA) to estimate that we would have 82%–93% power (depending on absolute amounts of absorbed zinc) to detect a difference of 0.2 mg of daily absorbed zinc between the 3 complementary food groups with 15 subjects per group. This amount of absorbed zinc represents approximately 25% of the estimated physiologic requirement for zinc at this age.

### 2.4. Randomization and Enrollment

Potential families were visited and educated on the proposed study before the enrollment visit and the study nurse or lab technologist obtained written informed consent from the parents/guardians plus witness upon confirmation that the child met the inclusion and exclusion criteria. Forty-five pieces of paper with letters marked A, B or C to represent the three study arms were rolled into balls and placed in a plastic container. The pieces were mixed thoroughly through shaking of the container. An independent person then picked the pieces one by one from the container. The unique number-letter became the subject and group assignment for each individual.

### 2.5. Intervention

The micronutrient content of the two MNP preparations included zinc (5 mg), Vitamin A (300 μg), Vitamin C (30 mg), folic acid (160 μg), and iron (12.5 mg or 0 mg). The powder for Group C was a product very similar to the other group powders without the additional micronutrients. All of the MNP sachets were marked with either A, B, or C by the manufacturer (Hexagon Nutrition Pvt. Ltd., Mumbai, India), and were otherwise indistinguishable. The code was known only to a senior investigator not involved in the field work (FE), thus assuring blinding of group assignment to the participating subjects and to the field research teams. Laboratory analysis at the University of Colorado verified the accuracy of the zinc and iron contents of the products.

The MNP were provided at weekly intervals during the 3-month intervention period from 6–9 months of age. At the end of each visit 7 sachets were left with the mother plus 3 extra in case a delay occurred in follow up visits. Mothers were instructed to give one sachet daily and to add the entire contents of the sachet to one meal. All children were followed up once a week by the nurse or clinical officer to ascertain if the MNP were taken during the 3 months of follow up and to replenish the next week’s supply of the MNP sachets.

### 2.6. Anthropometry

For descriptive purposes, length and weight were obtained on the infants at enrollment and at the end of the study period. Lengths were measured with a portable infantometer supplied by the Ministry of Health, Department of Nutrition; instrument was accurate to 0.5 cm. Weights were obtained with a weighing scale model RGZ-20 (Wuxi Weight Factory, Wuxi City, China), accurate to 50 g. The field team received extensive training prior to the initiation of the field studies. Measurements were taken in triplicate at the village elders’ homes where the infantometer and scale were maintained [21].

### 2.7. Zinc Absorption Study

The isotope studies were undertaken when the participating infants were approximately 9 months of age. Baseline stool and urine specimens were taken prior to isotope dosing. Weighed doses of oral stable isotope ^67^Zn were administered on day 1 with the MNP meal (primarily ugali maize porridge). Weighed doses of ^70^Zn were administered with all other meals (excluding breast milk). For both isotopes, the doses were based on the estimated amount of zinc in the meals (non-MNP and MNP), with the total dose representing approximately 10% of daily zinc intake (estimated to be ~6–7 mg/day for the MNP groups and 1–2 mg/day for the control group). Thus, the isotope doses were the same for the two MNP groups containing zinc and lower for the placebo MNP preparation. The isotope preparations were indistinguishable except for labeling, to maintain the blinding of the MNP group assignment. After the last meal of the day, the dose of ^68^Zn was administered intravenously into an antecubital vein. Infusion of the isotope dose was followed by infusion of 2 rinses of normal saline to assure administration of the complete dose. Extensive training and supervision of the isotope administration and specimen collections were provided by the UCD team (Diana Culbertson, Nancy F. Krebs and K. Michael Hambidge). A manual of operations was developed for all procedures and availed to field personnel. All supplies for the isotope studies were metal-free and provided by the UCD team.

#### 2.7.1. Isotope Dose Preparation and Administration

Enriched stable isotopes of zinc were obtained from Trace Science International (Richmond Hill, Canada). Accurately weighed quantities of each isotopically enriched preparation were dissolved in 0.5 mol/L H_2_SO_4_ and then diluted with deionized water to prepare stock solutions. To prepare the oral doses, the stock solutions of ^70^Zn (95.5% enrichment) and ^67^Zn (94.2% enrichment) were diluted with purified water and titrated to a pH 3.0 with metal-free ammonium hydroxide. Zinc concentrations of the solutions were determined by triplicate measure of the preparation using atomic absorption spectrophotometry, adjusting the concentration measurements for the different atomic weights of the preparations. To prepare the intravenous dose, a stock solution of ^68^Zn (99.4% enrichment) was diluted with 0.45% saline, adjusted to pH 6.0 and then filtered through a 0.22-μm filter to ensure sterility. The pharmaceutical quality of the sterile solution (*i.e.*, sterility and pyrogenicity) was certified by the University of Colorado Hospital pharmacy and the core laboratory of the General Clinical Research Center, respectively.

Solutions of the ^70^Zn and ^67^Zn were given as small sips, starting at the mid-point of each meal, alternating between bites of the foods and the labeled solution. All losses of doses from drooling or spits were collected on ashless filter paper during the dose administration. These were analyzed using ICP-MS (inductively coupled plasma mass spectrometry) and the amount of isotope lost during administration was subtracted from the calculated dose to give a final administered isotope dose. Intravenous infusion of ^68^Zn was undertaken through a “butterfly” needle and tubing; a 3-way stop-cock was used to allow infusion through one port and rinsing of isotope syringe through the other port. Prior to infusion of the isotope dose, blood for the ancillary biomarkers was obtained.

#### 2.7.2. Sample Collections

Weighed duplicate diets were collected for the entire day of the isotope studies and transferred to polypropylene containers. Approximately 20 mL urine samples were collected twice a day (a.m. and p.m.) for days 4 to 7 after the oral isotope administration. Urine was collected using standard zinc-free pediatric urine collection bags, and subsequently transferred to clean polypropylene containers. Diet and urine specimens were frozen at −20 °C until shipment to UCD.

#### 2.7.3. Sample Processing and Analysis

Duplicate diets were dried in an electric drying oven before ashing at 450 °C; samples were reconstituted in 6 M HCl and total zinc concentration was measured using atomic absorption spectrophotometry fitted with a deuterium arc background lamp (model AAnalyst 400, Perkin-Elmer Corporation, Norwalk, CT, USA) [22]. Urine samples were digested using a MARS microwave sample preparation system (CEM Corp, Matthews, NC, USA), as previously described [22]. Isotope enrichment was determined by measurement of the isotope ratios ^67^Zn/^66^Zn, ^68^Zn/^66^Zn and ^70^Zn/^66^Zn by inductively coupled plasma mass spectrometry (Agilent 7700x ICP-MS, Agilent Technologies, Santa Clara, CA, USA) and conversion of ratios to enrichment using a mathematical matrix. Tracer enrichment was defined as all of the zinc in the sample that was derived from the isotopically enriched tracer preparation divided by the total zinc in the sample [23].

### 2.8. Biomarkers 

Blood was obtained at 6 and 9 months for serum zinc, biomarkers of iron status (ferritin and soluble transferrin receptor (sTfR)), and systemic inflammatory markers α 1-acid glycoprotein (AGP) and C-reactive protein (CRP). A portable field based hemoglobin analyzer (HemoCue 201^+^, HemoCue America, Brea, CA, USA) was used for screening hemoglobin measurements, and a Partec Malaria Cyscope machine (Partec East Africa Ltd., Nairobi, Kenya) was used for malaria screening. Blood was centrifuged at the homestead using a handheld centrifuge (Hettich Hand-zentrifuge 1011, Tuttlingen, Germany). Syringes and storage tubes were all zinc free, and precautions were taken in the field to avoid zinc contamination. The specimens were placed on ice at the home visits, and were held in a −20 °C freezer for overnight stay at the local hospital until transport to the −70 °C freezer at Moi University the next day using an ice packed vaccine carrier. Serum zinc was analyzed by atomic absorption spectrophotometry with background correction, and AGP by R & D Systems Quantikine Enzyme-linked Immunosorbant Assay (ELISA); both were run in the Pediatric Nutrition Laboratory at UCD. Serum ferritin, sTfR, and CRP were analyzed by nephelometry in the Pediatric Clinical Translational Research Center core laboratory at UCD.

### 2.9. Data Processing and Statistical Analyses

#### 2.9.1. Data Processing

Paper data forms were developed for all activities and biospecimen collections. Data entry was conducted in Eldoret into a secure, web-based data management system (REDCap, Research Electronic Data Capture, supported by the Colorado Clinical Translational Science Institute at UCD), and was accessible to only registered research team members at UCD, Indiana University and Moi University.

The total daily dietary intake of zinc from all non-breast milk foods at the time of the metabolic studies was determined from the zinc content in the duplicate diet. Fractional absorption of zinc (FAZ) was measured by the DITR in urine method [16]. Specifically, FAZ = enrichment (oral/intravenous) × dose (intravenous/oral). Daily “total” absorbed zinc (TAZ, mg/day) from all non-breast milk foods was calculated by multiplying daily dietary zinc intake (mg) by FAZ. The EZP, defined as the estimate of the total size of the combined pools of zinc that exchange with zinc in plasma within approximately 2–3 days, was calculated by dividing the dose of intravenous isotope (^68^Zn) infused by the enrichment value at the y-intercept of the linear regression of a semi-log plot of urine enrichment data from study days 5–8 after isotope administration [17].

#### 2.9.2. Statistical Analyses

Results were analyzed and plots created using GraphPad Prism version 6 (GraphPad Software, San Diego, CA, USA). The R statistical programming environment was also used for statistical analysis [24]. Group data sets were examined for normality and homoscedasticity. To minimize the impact of outliers and skewed distributions, 12.5% trimmed means were used in the equality of means testing of the zinc isotope data. One-way ANOVA and Tukey’s honest significant difference (HSD) multiple comparisons test were used when data were normally distributed and exhibited equal variances. When variances were not equal, Welch’s F test and the Dunnett-Tukey-Krammer multiple comparisons test were used to compare means. Repeated measure ANOVA was used to assess the effect of time and group for growth and biochemical assays. Data are presented as mean (SEM) unless otherwise noted. Significance was defined at α = 0.05 level.

## 3. Results

No differences in demographic or anthropometric measurements were observed among the study groups at either baseline or end of the intervention. Weight and length both significantly increased over the study period (Table 1). No cases of malaria were identified in the subjects during the study period. Compliance monitoring for the assigned MNP indicated >90% consumption among all of the participants.

**Figure 1:**
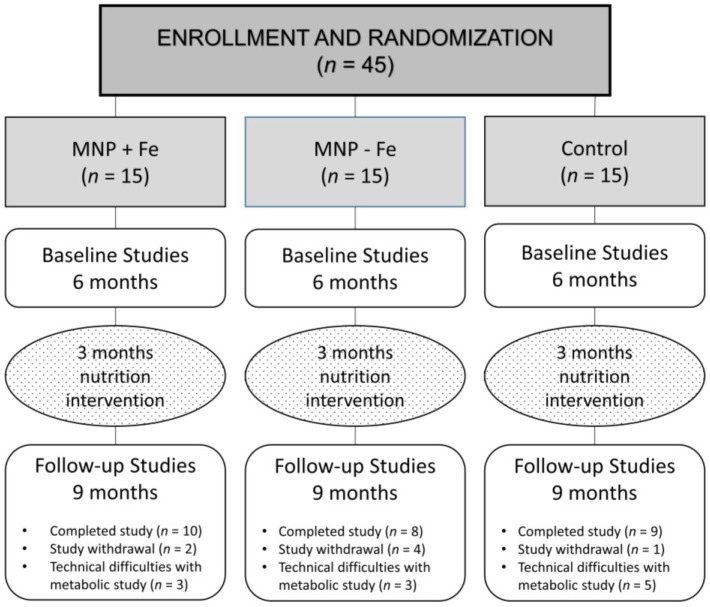
Consort diagram. MNP, micronutrient powder.

Isotope studies were successfully completed on 10, 8, and 9 subjects in the MNP + Fe, MNP − Fe, and Control groups, respectively. Reasons for failure to complete the isotope studies included participant withdrawal from the study during the intervention stage (*n* = 7) or technical difficulties with isotope administration and/or incomplete sample collections (*n* = 11) (Figure 1).

**Table 1:** Demographic and anthropometric data at 6 and 9 months ^1^.

	6 months of Age			9 months of Age		
	MNP + Fe (*n* = 15)	MNP − Fe (*n* = 15)	Control (*n* = 15)	MNP + Fe (*n* = 10)	MNP − Fe (*n* = 8)	Control (*n* = 9)
**Gender**						
Male	5 (33%)	7 (47%)	10 (67%)	3 (30%)	3 (37%)	7 (78%)
Female	10 (67%)	8 (53%)	5 (33%)	7 (70%)	5 (63%)	2 (22%)
**Weight, *kg*^2^**	7.25 (0.25)	7.55 (0.27)	7.49 (0.22)	8.17 (0.32)	8.2 (0.44)	8.37 (0.36)
**WAZ ^3^**	−0.39 (0.25)	−0.16 (0.31)	−0.21 (0.25)	−0.36 (0.32)	−0.33 (0.45)	−0.38 (0.41)
**Length, *cm*^2^**	65.0 (0.7)	66.5 (0.9)	64.6 (0.8)	67.9 (1.2)	70.2 (1.7)	69.5 (2.0)
**LAZ ^3^**	−0.68 (0.27)	−0.19 (0.40)	−1.01 (0.39)	−1.16 (0.39)	−0.21 (0.83)	−0.71 (0.86)

^1^ Mean (SEM); ^2^ repeated measure ANOVA showed no difference between groups at 6 months or 9 months, significant increase over time for both weight (*p* < 0.01) and length (*p* < 0.05) without group effect; ^3^ repeated measure ANOVA showed no difference between groups at 6 months or 9 months, no significant effect of time or group. MNP, micronutrient powder; WAZ, weight-for-age *Z*-score; LAZ, length-for-age *Z*-score.

**Figure 2:**
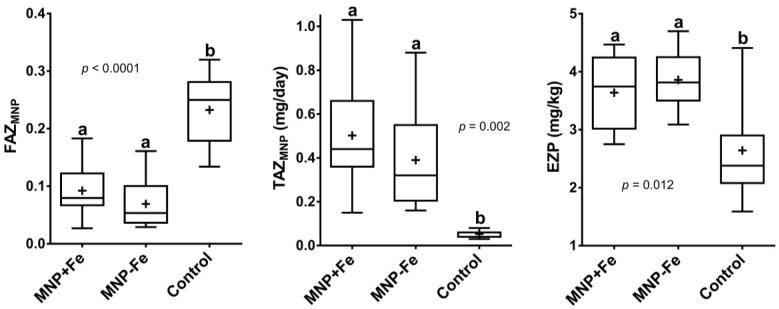
Box and whisker plots of trimmed mean data for (**A**) fractional absorption of Zinc (FAZ) from micronutrient powder (MNP) meals; (**B**) total absorbed zinc (TAZ) from MNP meals and (**C**) exchangeable zinc pool (EZP) after 3 months on intervention. “+” indicates mean value. Different superscripts denote significant differences between groups (ANOVA).

Equality tests of the trimmed means of daily zinc intake, FAZ, TAZ, and EZP all indicated significant differences. As expected, the MNP provided a large increase in daily zinc intake over that consumed from the usual diets, represented by the C group. Due to the large difference in variance between the control and intervention groups, the Welch test was used to compare the zinc intake means, resulting in *p* < 0.0001. One-way ANOVA detected significant differences in the mean FAZ and TAZ (Welch test) from the MNP meals and in EZP. For intake, FAZ, TAZ, and EZP, the post-tests on the trimmed data showed that the MNP + Fe and MNP − Fe groups were not different and that the control group was different from either intervention group (Figure 2).

Data from the zinc absorption studies in all subjects are summarized in Table 2.

**Table 2:** Untrimmed data for zinc intake from complementary foods with and without micronutrient powder (MNP) added to the meal, fractional absorption, and total absorbed zinc by group ^1^.

	MNP + Fe (*n* = 10)	MNP − Fe (*n* = 8)	Control (*n* = 9)	*p*-value (ANOVA)
**Total Daily Zn Intake, *mg/day***	6.53 (0.22) ^a^	6.69 (0.28) ^a^	0.90 (0.11) ^b^	0.0001
**FAZ, non-MNP meals**	0.22 (0.04)	0.20 (0.04)	0.27 (0.01)	0.41
**FAZ, MNP meals**	0.12 (0.04) ^a^	0.09 (0.03) ^a^	0.25 (0.04) ^b^	0.01
**TAZ ^2^, *mg/day***	0.85 (0.22) ^a^	0.72 (0.19) ^ab^	0.24 (0.03) ^b^	0.04

^1^ Mean (SEM) different superscripts within a row denote significant differences (*p* < 0.05); ^2^ sum of absorbed zinc from non-MNP meals +MNP meals. FAZ, fractional absorption of Zinc; TAZ, total absorbed zinc.

Results of hemoglobin, serum zinc and EZP, and inflammatory markers for each group at the beginning and end of the study are presented in Table 3. No difference was observed by group or time for any of the iron biomarkers except sTfR, which showed a significant increase over time (*p* = 0.02) and a trend of group-by-time interaction (*p* = 0.09) for the increase that occurred in the placebo and the MNP − Fe group. Means for CRP were above the upper limit of the normal range for all groups at both time points. In addition, plasma Zn concentration significantly decreased over time (*p* = 0.008) without any group effect. Mean EZP of all data, measured after three months on intervention, did not differ by group.

**Table 3:** Biomarkers at 6 and 9 months ^1^.

Biomarker	MNP + Fe	MNP − Fe	Control
Hemoglobin, *g*/*dL*			
6 months	11.3 ± 0.8 (10)	12.4 ± 1.0 (8)	12.0 ± 0.5 (9)
9 months	12.0 ± 0.4 (10)	14.3 ± 0.9 (4)	12.8 ± 0.5 (8)
Plasma Zn, *μg*/*dL* ^2^ (60–110)			
6 months	80.8 ± 1.0 (8)	78.3 ± 7.1 (6)	81.1 ± 6.4 (9)
9 months	70.2 ± 5.6 (8)	69.8 ± 5.4 (6)	62.2 ± 4.2 (9)
Ferritin, *μg*/*L* (17–145)			
6 months	46.9 ± 9.8 (8)	25.4 ± 6.7 (6)	38.7 ± 6.7 (9)
9 months	49.9 ± 12.7 (8)	26.5 ± 10.9 (6)	23.7 ± 11.1 (9)
Serum transferrin receptor (sTfR), *mg*/*L* ^3^ (0.76–1.76)			
6 months	3.0 ± 0.4 (10)	2.4 ± 0.2 (8)	2.8 ± 0.1 (9)
9 months	2.8 ± 0.3 (10)	3.1 ± 0.4 (8)	4.0 ± 0.4 (8)
Α 1-acid glycoprotein (AGP), *mg*/*dL* (50–120)			
6 months	87.4 ± 14.1 (10)	81.0 ± 17.3 (7)	81.6 ± 14.5 (9)
9 months	90.9 ± 19.3 (10)	80.8 ± 11.5 (7)	79.7 ± 12.9 (9)
C-reactive protein (CRP), *mg*/*L* (<3)			
6 months	7.7 ± 4.5 (10)	11.5 ± 5.9 (8)	5.3 ± 1.8 (8)
9 months	6.2 ± 3.4 (10)	8.3 ± 4.2 (8)	6.2 ± 1.7 (9)
Exchangeable zinc pool (EZP), *mg/kg*			
9 months	3.7 (0.4)	4.0 (0.4)	3.0 (0.6)

^1^ Mean ± SEM (*n*), repeated measure ANOVA was used to assess the effect of group and time (6 months *vs.* 9 months), normal range in parentheses follows biomarker heading; ^2^ effect of time (*p* = 0.008): plasma Zn concentration decreased over time without differences between groups; ^3^ effect of time (*p* = 0.02): sTfR increased over time; A trend of time-by-group interaction was observed (*p* = 0.09): sTfR increased in MNP − Fe and Control groups but not in MNP + Fe group. MNP, micronutrient powder.

## 4. Discussion

The results of this study do not support an adverse effect of the fortificant iron from MNP on zinc absorption in this group of infants in a malaria endemic area. This is consistent with others’ conclusions that iron fortification of a meal does not adversely affect zinc absorption [13,14,25]. An additional important finding, however, is the lower than expected zinc absorption, regardless of iron content of the MNP fortified meals.

Despite a substantially increased zinc intake compared to usual intakes for this community (represented by the placebo group) and relative to the EAR of 2.5 mg/day for this age, the amount of daily absorbed zinc (TAZ) was at or below the estimated physiologic requirement of 0.84 mg/day [26]. Similar fractional absorption from maize-based test meals (without an extended MNP intervention) was reported by Zlotkin *et al.* for infants in Ghana who received MNP containing 30 mg of iron and either 5 or 10 mg of zinc. In that study infants, only the high zinc preparation resulted in absorbed zinc at the estimated physiologic requirement, whereas the mean absorption from the 5 mg preparation was only 0.3 mg, even less than observed in the current study [27]. By comparison, nine-month-old breastfed infants in Denver achieved daily absorbed zinc near the physiologic requirement from complementary foods with average zinc intakes that averaged only about 60% of the intakes for the Kenyan infants receiving the zinc containing MNP [22]. The conclusion that the absorption of zinc was impaired in the present study is also supported by examination of the data from other groups of young children in low resource settings, for whom the amount of absorbed zinc was similar to that achieved in the current study despite lower dietary zinc intakes [28,29].

Although the dietary staple maize, consumed primarily as a liquid porridge, is high in phytate, the zinc from the MNP would have substantially lowered the phytate-zinc molar ratio. We did not measure intake and absorption from breast milk, but the intake from this source at nine months is likely to be less than 0.5 mg per day [30]. Fractional zinc absorption of 0.50 from human milk would be expected [22,31]. If this was achievable in this setting, the contribution to physiologic requirements, though modest, could be important.

Markers of intestinal permeability and enteropathy were not obtained in this population, but this is a setting and age group in which environmental enteropathy may be common and provide a plausible explanation for the poor zinc absorption. Environmental enteropathy is considered to have a negative impact on small intestinal function and absorptive capacity [32]. Furthermore, if enteropathy was present in these infants, intestinal endogenous zinc losses may also have been relatively high [33] and would thus lead to even higher requirements for absorbed zinc.

The primary determinants of zinc absorption for healthy adults are amount of zinc ingested and dietary phytate [34]. For infants and children, the determinants are less clear but may also include gut length or age [35]. The effects of inflammation on absorption have not been systematically examined in either adults or children. Whether due to enteropathy or other factors, these infants seemed to exhibit an impaired homeostatic response. The significant decline in plasma zinc concentrations for all groups suggests a failure to improve zinc status despite the intake from MNP. After three months of a generous intake, zinc transporters might be expected to be saturated and “down regulated” [34]. In the face of potentially relatively high requirements, however, down-regulation would be un-physiologic or maladaptive. The placebo group predictably had a higher fractional absorption of zinc, but with such low dietary zinc intakes, this response was notably inadequate to compensate to meet zinc physiologic requirements. These results in totality emphasize the limits of absorptive mechanisms to compensate for adverse conditions, whether due to impaired gut health, immunostimulation, or limited dietary intake.

The EZP sizes were also low relative to the dietary intakes and compared to Denver breastfed infants of the same age and with lower dietary zinc intakes [22]. Specifically, despite dietary zinc intakes of the MNP groups that were considerably higher than those of Denver infants receiving regular meat or a zinc fortified cereal, the EZP sizes of the two MNP groups in the present study were only ~85% of the mean EZP sizes for the Denver infants [22]. This, along with the decline in plasma zinc concentrations, further supports chronically impaired Zn absorption. Additionally, the mean EZP for the placebo group was below our tentative lower cut-off of 3 mg/kg for this age [36]. 

The effect of the MNP + Fe on iron status was at best modest, but the small sample size and high inflammatory burden greatly complicate the interpretation of the biomarker results. Despite eligibility criteria including only mild anemia, and the finding of normal mean ferritin levels at the beginning of the intervention at six months, normal iron status cannot be assumed due to the high levels of inflammatory markers, including elevated CRP levels for most subjects. The sTfR values of virtually all subjects exceeded the upper limits of normal range at baseline, and were above normal range for most subjects at nine months, including for the infants who received the MNP + Fe. In addition to presumed negative effect of high phytate diet on iron absorption, the elevated inflammatory state could reasonably be expected to also stimulate hepcidin, which would further impair absorption, even in the face of iron deficiency [37]. The significant decline in plasma zinc concentrations between six and nine months for all groups may also reflect the overall large inflammatory burden, but the small sample size warrants cautious interpretation. A recent review of iron supplementation found that administration of iron and zinc supplements together resulted in significantly reduced zinc concentrations compared to zinc supplements given alone [38]. The iron dose used in the MNP + Fe group is similar to that used in other studies of MNP’s, including the study in Tanzania in which iron supplementation in iron-replete subjects was associated with more infectious morbidity [1] and in a recent study in which assignment to MNP with iron was associated with more frequent bloody diarrhea [39]. Emerging data suggest potential for fortificant iron to induce pro-inflammatory changes in the intestine and the microbiota [40,41,42,43] but evidence for iron-associated systemic inflammation with fortificants is less clear. The lack of effect of iron on zinc absorption and the pattern of changes in biomarkers suggests an interaction between these two essential trace elements systemically, rather than competition for transport by the enterocyte.

The notable limitations of this study relate to the smaller than expected sample size for successful completion of the demanding zinc metabolic studies. With this limitation, the possibility of failing to detect an effect of the iron on zinc absorption due to low statistical power must be considered. However, the similarity of the EZP sizes between the two MNP groups, and the significantly higher EZP means compared to that of the control group, does not suggest an iron effect on zinc absorption. The strikingly high inflammatory burden also greatly increased variability and impacted the ability to detect differences in micronutrient status among groups. Although the degree of systemic inflammation suggests underlying enteropathy, the lack of markers of intestinal inflammation does not allow confirmation of the underlying condition. The strengths of the study were the randomized design; the longitudinal follow-up; and the measurements of zinc absorption directly in a population at high risk for both zinc and iron deficiencies, for malaria, and for environmental enteropathy.

## 5. Conclusions

No effect of iron on zinc absorption was apparent in this malaria endemic setting when iron was given as a component of a MNP. Despite a substantial increase in zinc from the MNP, which brought their habitual dietary zinc intakes to double the estimated average requirement for a duration of three months, the mean daily absorbed zinc for both MNP groups barely achieved the estimated physiologic requirement for this age [26,44,45]. Furthermore, the size of the EZP for both MNP groups was lower than observed for breastfed infants in more hygienic settings who had lower habitual zinc intakes [22]. All of these observations are consistent with impaired zinc absorption in these breastfed infants at risk for zinc deficiency as well as for environmental enteropathy.
